# Unexpected tracheal agenesis with prenatal diagnosis of aortic coarctation, lung hyperecogenicity and polyhydramnios: a case report

**DOI:** 10.1186/s13052-020-00861-0

**Published:** 2020-07-10

**Authors:** Alessandro Perri, Maria Letizia Patti, Annamaria Sbordone, Giovanni Vento, Rita Luciano

**Affiliations:** 1 Department of Woman and Child Health and Public Health, Child Health Area; Fondazione Policlinico Universitario A. Gemelli, IRCCS, Università Cattolica del Sacro Cuore, Largo A. Gemelli 8, 00168 Rome, Italy; 2 Department of Woman and Child Health and Public Health, Child Health Area, Università Cattolica del Sacro Cuore, Roma, Italy

**Keywords:** Agenesis, Trachea, Congenital malformations, Airway obstruction

## Abstract

**Background:**

Tracheal agenesis (TA) is a rare disorder usually diagnosed prenatally when a congenital high airway obstruction syndrome (CHAOS) is identified. We present a case of unexpected TA in a neonate without prenatal diagnosis of airway obstruction, with a difficult management at birth. Moreover, we discuss about differential diagnosis, classification and treatment issues.

**Case presentation:**

A 2280 g female neonate was born at 35 week gestational age (GA) with prenatal diagnosis of aortic coarctation, polyhydramnios and diffuse hyperechogenicity of the right lung. At birth, the neonate had no audible cry, no air entry to the lungs, and hypotonia. Tracheal intubation was unsuccessful, and no visualization of the trachea was obtained when tracheostomy was attempted. Post-mortem examination showed tracheal agenesis associated with tracheoesophageal fistula and revealed no cardiologic malformations. Aortic coarctation had been suspected prenatally because of the first portion of the descendent thoracic aorta being compressed by a fibrous band connecting the proximal and distal tracheal branches. CHAOS had not developed due to the tracheoesophageal fistula (TOF).

**Conclusions:**

TA is not always diagnosed in the fetus and it may present unexpectedly making the neonate’s management at birth critical. An effective rescue temporary oxygenation may be obtained with mask ventilation or oesophageal intubation in those cases of TA associated with a TOF. We suggest to consider a fetal magnetic resonance imaging (MRI) when the association polyhydramnios/lung hyperechogenicity occurs, even in the absence of CHAOS or other malformations. Once a diagnosis is provided, the mother should be transferred to selected centres where an ex-utero intrapartum procedure (EXIT) can be attempted. Moreover, despite high mortality, different surgical management are described to improve survival.

## Background

TA is a rare congenital disorder with an incidence of about 1:50.000 births [1, 2] and affect more male than female [3]. It is usually diagnosed prenatally because a CHAOS occurs. In presence of TOF, CHAOS doesn’t develop and the diagnosis is made at birth when the neonate shows a severe respiratory distress that is shortly followed by cardio-respiratory arrest [4]. When a TOF is associated with TA, polyhydramnios and diffusely increased echogenicity of the lungs are the only signs possibly detected by prenatal ultrasonography [5, 6]. Different theories have been suggested to explain the causes of tracheoesophageal anomalies. Many genetic defects are associated with incorrect separation of the foregut, and a major role for the bone morphogenetic protein (BMP) type I receptor genes in mouse models was suggested [7]. No causal gene has been identified in human TA patients yet. TA can occur alone or in association with multiple anomalies. It may occur as part of vertebral defect, anal atresia, radial dysplasia, renal defects and cardiovascular defects association (VACTERL) or as part of tracheal agenesis/atresia, complex congenital heart disease, radial ray defects, and duodenal atresia (TACRD) syndrome [8] **.**

This rare congenital defect is often incompatible with life. Survival after delivery is dependent on prompt diagnosis and airway management [3]. Nowadays prenatal diagnosis of TA is critical to give a chance of survival to the neonate, even though the ideal surgical therapy for TA is still not defined and prognosis after treatment is poor [6]. Prenatal MRI may provide a definitive diagnosis of TA and should be considered in case of polyhydramnios associated with other congenital malformations.

We present a case of unexpected TA in delivery room, without prenatal diagnosis of CHAOS, but evidence of aortic coarctation, lung hyperechogenicity and polyhydramnios at fetal ultrasonography.

## Case presentation

**Fig. 1:**
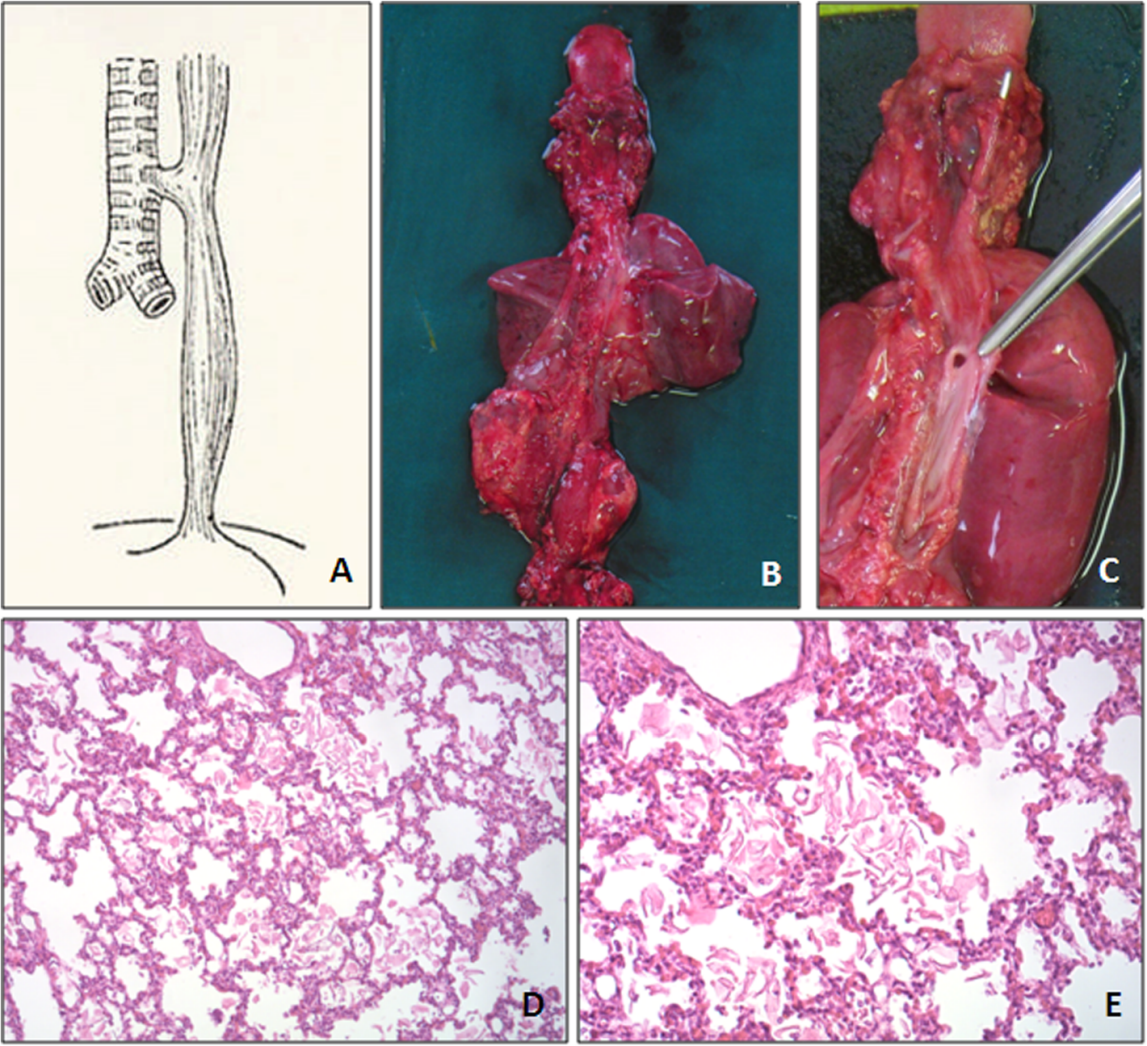
**a**, Tracheal Agenesis with an upper trachea joined by atresic strand to a distal trachea that has a fistulous connection with the esophagus (classification of tracheal agenesis by Faro et al., type D). **b**, Postmortem specimen: pulmonary architecture and lungs development are normal. **c**, Upper trachea with its proximal blind end (arrow). Lower trachea opens directly into midesophagus (arrowhead). **d-e**, Filling of alveolar ducts and alveolar lumens with squames and squamous cells. Capillary congestion is present

A 2280 g female neonate was born at 35 week gestational age (GA). Aortic coarctation was suspected at the 22 week GA ultrasound and confirmed by fetal echocardiography 1 week later. The ultrasound performed at 35 of gestation weeks showed diffuse hyperechogenicity of the right lung that was misdiagnosed as cystic adenomatous malformation. At the same time, severe polyhydramnios was diagnosed and treated with amnioreduction. Following this procedure, premature rupture of membranes and contractile activity occurred. Emergency caesarean section was then performed according with the fetal aortic coarctation. The neonate presented with no audible cry, gasps, no air entry to the lungs and hypotonia. Positive pressure mask ventilation was started, that did not result in thoracic excursions. Several attempts of intubation were performed but the tube did not progress beyond the vocal cords even though the glottis could be clearly visualised. Stabilization with mask ventilation at 100% oxygen was obtained. At 10 min of life, heart rate was about 140 bpm and SatO2 was about 85–90%, while gasping, no audible cry and hypotonia persisted. APGAR score was 2–5-5, respectively at 1, 5 and 10 min. Umbilical venous blood sample, obtained at 30 min of life, showed severe respiratory acidosis: pH 6.85, PCO2 147 mmHg, and base-excess was − 11.3 mmol/L. Tracheostomy attempted by the otorynolaryngologist was impossible. Clinical conditions got worse and severe bradycardia occurred. Resuscitation was performed, consisting in thoracic compression and administration of repeated doses of IV adrenalin (0.1 ml/kg adrenalin 1:10.000), that only resulted in transient rise of heart frequency to 80 bpm. Despite sustained resuscitative efforts the baby died at 150 min of life. Post-mortem examination (Fig. 1) showed a normal larynx and a proximal tracheal stump that ended in a blind pouch 0.4 cm below the vocal cords. A 1.3 cm distal stump of trachea with a proximal blind end was also founded. The two tracheal stumps were separated by a fibrous band whose length was 5.3 cm. This fibrous band was located ahead the first portion of the descendent thoracic aorta. From the distal tracheal stump arose bronchi of normal calibre. Lower trachea opened directly into midoesophagus. Pulmonary architecture was normal, as was lungs development. Alveolar ducts and alveolar lumens were filled with squamous cells. Capillary congestion was present. These findings are consistent with a D variant of Faro classification of TA. No other malformations were founded.

## Discussion and conclusions

TA is a complete or almost complete absence of the trachea, with or without TOF. The most popular classification of TA is the Floyd classification [9] that describes three types of TA. Type I has agenesis of the proximal trachea and a short distal tracheal remnant with a tracheoesophageal fistula. Type II has the complete agenesis of the trachea, and the bronchi communicate at the carina with a tracheoesophageal fistula. Type III has complete agenesis of the trachea, and the bronchi communicate through separate fistulas with the distal oesophagus. In 1979, Faro et al. [10] proposed a more complex classification of TA in seven types: A, total pulmonary agenesis; B, TA with main bronchi arising directly from oesophagus; C, TA with fused main bronchi and bronchoesophageal fistula; D, TA with larynx joined by an atresic strand to the distal trachea that has a fistulous connection with the oesophagus; E, upper TA with large direct tracheoesophageal communication; F, agenesis of the proximal trachea with a normal caudal segment of the distal trachea and no TOF; G, short segment TA. Our patient had the features of type D according to Faro classification.

The diagnosis of TA may be established prenatally if a CHAOS occurs. The syndrome presents with bilaterally enlarged echogenic lungs, which are due to intrapulmonary liquid retention caused by tracheal agenesis. Flattened diaphragm and ascites can be observed. In our case the prenatal diagnosis of tracheal agenesis was not evoked because the TOF preserved the fetus from CHAOS. In fact, the presence of associated TOF allowed the evacuation of intrapulmonary liquid and therefore the CHAOS was not present. A fibrous band connecting proximal and distal tracheal branches compressed the first portion of the descendent thoracic aorta, thus leading to the erroneous diagnosis of aortic coarctation.

When TOF is present, unexpected polyhydramnios, due to defective fetal swallowing, and lung hyperechogenicity, especially when associated with other congenital malformations, should alert clinicians of potential tracheal anomalies. In those conditions, a fetal MRI might establish a definitive prenatal diagnosis allowing the physicians to inform the parents and consider treatment strategies. Multidisciplinary management may be necessary. That includes the opinions of neonatologist, obstetrician, anaesthetist, otolaryngologist and paediatric surgeon. Therefore, once a diagnosis is provided, the mother should be transferred to selected centres where an EXIT strategy could be attempted. In cases without TOF even initial stabilization is impossible and the condition is invariably fatal [3]^.^ Therefore the diagnosis of tracheal agenesis with oesophageal fistula, even in the absence of CHAOS, assumes a crucial role as a correct perinatal management offers a chance of survival. In the case of TA with TOF, mask ventilation or oroesophageal intubation can provide effective rescue temporary oxygenation (as happens in our case) until ECMO is started. The surgical approach, then, is not necessarily needed immediately after birth [11], but a limited number of cases treated with a surgical management are reported [6, 11, 12]. Most treated and survivors patients could not be weaned off mechanical ventilation and/or suffered from neurological impairment because of the difficulty in achieving long-term airway maintenance or owing to the lack of an established effective treatment method [3, 13]. A recent review, however, reported described four patients with TA surgically treated with oesophageal reconstruction that did not require permanent mechanical ventilation and whose development was close to normal [14]. All of them managed to communicate, vocalizing, with their families and doctors. Neurological development was normal in one of these cases and slightly delayed in two cases [14]. An innovative surgical approach, consisting in a stem cell based tissue engineered tracheal replacement, was also described [15] for the treatment of tracheal stenosis.

TA can be diagnosed at birth when orotracheal intubation is impossible to perform in neonates presenting with unexpected severe respiratory distress, hypotonia and no audible cry.

We present this case to underline that the absence of a prenatal diagnosis makes the management of TA at birth very difficult. Moreover, the real challenge is the suspicion of TA when a complete airway obstruction not occurs and when prenatal ultrasound findings are not clear or indicative of other major malformation. This is particularly relevant because infants with this presentation are the ones who have chance of survival if a prompt management with airway stabilization with EXIT.

In conclusion we suggest to perform a fetal MRI prenatally when the association of polyhydramnios and lung hyperechogenicity occurs, even in the absence of other malformations. If TA is diagnosed, multidisciplinary management should be performed in order to propose the parents the best treatment options.

## Data Availability

Not applicable.

## Abbreviations

TA: Tracheal Agenesis
CHAOS: Congenital High Airway Obstruction Syndrome
GA: Gestational Age
TOF: Trache-Oesophageal Fistula
BMP: Bone morphogenetic protein
VACTREL: Vertebral defects, anal atresia, tracheoesophageal fistula with oesophageal atresia, cardiovascular defects, and radial or renal dysplasia, plus and limb defects
TACRD: Tracheal agenesis/atresia, complex congenital heart disease, radial ray defects, and duodenal atresia
EXIT: Ex Utero Intrapartum Treatment
MRI: Magnetic Resonance Imaging
IV: Intravenous
ECMO: Extracorporeal membrane oxygenation
