# Correction to: Unexpected tracheal agenesis with prenatal diagnosis of aortic coarctation, lung hyperecogenicity and polyhydramnios: a case report

**DOI:** 10.1186/s13052-020-00879-4

**Published:** 2020-08-14

**Authors:** Alessandro Perri, Maria Letizia Patti, Annamaria Sbordone, Giovanni Vento, Rita Luciano

**Affiliations:** 1 Department of Woman and Child Health and Public Health, Child Health Area, Fondazione Policlinico Universitario A. Gemelli, IRCCS, Università Cattolica del Sacro Cuore, Largo A. Gemelli 8, 00168 Rome, Italy; 2 Department of Woman and Child Health and Public Health, Child Health Area, Università Cattolica del Sacro Cuore, Roma, Italy

**Correction to: Ital J Pediatr 46, 96 (2020)**

**https://doi.org/10.1186/s13052-020-00861-0**

The original article [1] contains two errors in Fig. 1:

1. Fig. 1A mistakenly shows a tracheoesophageal fistula instead of the intended image of a tracheal agenesis.
2. There is a small omission in Fig. 1C whereby arrows intended to annotate the image are missing.

The correct versions of Figs. 1A and 1C can be viewed ahead in this correction article.


